# The Clinical Effect of Endoscopic Mucosal Needle Aspiration Combined with Anhydrous Alcohol in the Treatment of Pneumatosis Cystoides Intestinalis

**DOI:** 10.5152/tjg.2026.25322

**Published:** 2026-05-18

**Authors:** Tingxu Yang, Wenjuan Feng, Yan Ma, Haixia Wang

**Affiliations:** Department of Gastroenterology, Shanghai First People’s Hospital Jiuquan Hospital, Gansu, China

**Keywords:** Absolute alcohol, colon, endoscopic treatment, pneumatosis cystoides intestinalis

## Abstract

**Background/Aims:**

To evaluate the clinical efficacy and safety of endoscopic mucosal needle aspiration combined with intracapsular anhydrous alcohol injection for colonic pneumatosis cystoides intestinalis (PCI).

**Materials and Methods:**

In this single-center, quasi-randomized comparative study, 86 patients diagnosed with colonic PCI and treated between April 2021 and April 2023 were allocated to a control group (needle aspiration alone, n = 43) or an observation group (needle aspiration plus alcohol injection, n = 43). Therapeutic efficacy, recurrence rate, complications, and postoperative satisfaction were compared between the two groups.

**Results:**

No immediate or short-term complications were observed, including bleeding, perforation, obstruction, or infection. Longer follow-up is needed to assess the late adverse events. At 3 months after treatment, the overall efficacy rate was significantly higher in the observation group (100%, 43/43) than in the control group (88.37%, 38/43; *χ*^2^ = 5.309, *P* = .021). No recurrence was observed in the observation group, whereas 5 cases (11.63%) recurred in the control group (*χ*^2^ = 5.309, *P* = .021). Furthermore, postoperative satisfaction was markedly higher in the observation group (95.35%) compared with the control group (79.07%).

**Conclusion:**

Endoscopic mucosal needle aspiration combined with intracapsular anhydrous alcohol injection was technically feasible. No immediate or short-term complications were observed. These findings suggest potential short-term benefit, but larger, multicenter studies with longer follow-up are needed to confirm effectiveness and late adverse events.

**Main Points**

- Endoscopic mucosal needle aspiration combined with intracapsular injection of anhydrous alcohol significantly improves the overall therapeutic efficacy of colonic pneumatosis cystoides intestinalis.
- The combined technique results in less postoperative recurrence compared to needle aspiration alone.
- No immediate or short-term complications were observed in either arm, including bleeding, perforation, or obstruction.
- Intracapsular alcohol injection produces cyst wall sclerosis, which improves the long-term therapeutic efficacy.

## Introduction

Pneumatosis cystoides intestinalis (PCI), also referred to as cystic lymphedema, cystic intestinal gas, or cystic intestinal wall pneumatosis, is an uncommon digestive disorder characterized by multiple submucosal or subserosal cysts filled with gas within the intestinal wall.^1^ The incidence of this condition is estimated to range from 0.03% to 0.2% in the general population,^2^ with the majority of lesions located in the colon. Epidemiological data indicate a higher prevalence in high-altitude regions such as Xinjiang, Qinghai, and Gansu, suggesting that environmental, dietary, and geographic factors may contribute to its development.^3^ While PCI can occur at any age, it is most commonly diagnosed in adults between 40 and 50 years old and shows a higher incidence in males.^4^

The exact pathogenesis of PCI remains unknown; nevertheless, hypotheses about the mechanisms have been offered, including mechanical, bacterial, pulmonary, and nutritional systems which hypothesize that gas can enter the intestinal wall due to micro-injury of the mucosa, bacterial fermentation, rupture of alveoli, or stasis in the lymphatic system.^5^^-^^8^ While each of the suggested mechanisms could account for PCI manifestations under specific circumstances, none can explain all of the clinical manifestations of PCI.^9^

Clinically, PCI often presents with nonspecific gastrointestinal symptoms such as abdominal distension, pain, diarrhea, and nausea. It is frequently associated with pre-existing conditions, including chronic obstructive pulmonary disease, connective tissue disorders, or gastrointestinal inflammation.^10^ While most cases of PCI follow a benign course, some can lead to serious complications such as intestinal obstruction, torsion, perforation with hemorrhage, or pneumoperitoneum. Severe complications may include intestinal wall perforation, resulting in acute abdomen and/or septic events that can become life-threatening.^11^^-^^13^

Conservative therapy, which includes oxygen inhalation, antibiotic treatment, bowel rest, and appropriate management of underlying conditions, is considered the first-line treatment for PCI.^14^^-^^16^ However, this approach is often ineffective, and recurrence is common even after an initial response. In recent years, endoscopic management has gained increasing recognition as a minimally invasive and effective alternative. The procedure allows for direct alleviation of cystic lesions within the intestine, providing symptomatic relief while aiming to reduce the likelihood of recurrence.^17^

Endoscopic needle aspiration has demonstrated promising short-term responses in case reports and small series. Takahashi et al^17^ published one case of PCI that responded to aspiration therapy alone. Wang et al^18^ reported successful endoscopic treatment in 6 patients. Tran et al^19^ and Molina et al^20^ offered further support that endoscopic intervention is clinically reasonable and safe; however, its subsequent use is limited in practice due to the recurrence of cystic lesions after conservative needle aspiration therapy.

To address the limitations of current treatments, several recent studies have explored adjunctive methods to enhance sclerotic changes in the cyst wall and reduce recurrence. Absolute (anhydrous) alcohol, a commonly used sclerosing agent, induces coagulation necrosis, endothelial destruction, and localized fibrosis when injected directly into the cyst. These effects promote closure of the residual cavity and prevent re-accumulation of gas. Despite its demonstrated clinical efficacy, evidence supporting its adjunctive use in PCI remains limited.

Given this gap in the literature, the present study aimed to provide further evidence on the clinical efficacy and safety of endoscopic mucosal needle aspiration combined with intracapsular (in-cyst) injection of anhydrous alcohol for the treatment of colonic PCI. It aimed to compare the outcomes between aspiration alone and aspiration with adjunctive alcohol injection, evaluating whether alcohol administration enhances cyst wall sclerosis, reduces recurrence, and improves patient satisfaction. The findings are intended to contribute new evidence to the growing body of knowledge on minimally invasive clinical approaches for PCI management.

## Materials and Methods

### Study Participants

A total of 86 patients diagnosed with PCI were treated at Shanghai First People’s Hospital Jiuquan Hospital between April 2021 and April 2023. Diagnosis was confirmed based on colonoscopic and computed tomography (CT) findings consistent with established PCI diagnostic criteria (Miwa et al^11^).

Given the relatively high detection rate of PCI in this region compared with other parts of China, regional clustering related to environmental, dietary, or altitude factors may partly explain the case volume.

Patients were allocated to a control group (n = 43) or an observation group (n = 43) using a quasi-random number table (1 : 1). There were no significant differences in baseline demographic or clinical characteristics between groups (*P* > .05), ensuring comparability.

The study was approved by the institutional Ethics Committee of Jiuquan People’s Hospital (Approval No.: 20210315; Date: March 15, 2021). All participants provided written informed consent prior to inclusion, and the study complied with the Declaration of Helsinki. The study was not prospectively registered in a public trial registry.

### Inclusion and Exclusion Criteria

#### Inclusion Criteria:

1. Diagnosis of PCI confirmed by colonoscopy and CT imaging consistent with established diagnostic standards^11^
2. Age ≥ 18 years
3. Ability to tolerate colonoscopy intervention
4. Provision of informed consent
5. Complete clinical and follow-up data.

#### Exclusion Criteria:

1. Severe multiple organ dysfunction (e.g., renal failure, heart failure, or hepatic failure)
2. Severe cardiovascular or cerebrovascular disease, epilepsy, or psychiatric disorder
3. Inability to tolerate colonoscopy or general anesthesia;
4. Voluntary withdrawal during the study
5. Previous participation in similar interventional trials.

Group allocation was performed using a random number table (1 : 1) in order of enrollment. Allocation concealment was not used. Given the lack of blinding and prospective trial registration, the study was best described as a single-center, quasi-randomized comparative study.

### Methods

All patients received bowel preparation with compound polyethylene glycol electrolyte powder before the procedure to ensure complete intestinal emptying. The colonoscope was advanced to the lesion, which was positioned in the 6 o’clock direction for optimal visualization.

Control group:Endoscopic mucosal needle aspiration was performed using a disposable mucosal injection needle (23G × 4 mm, Nanjing Nanwei Medical Co., Ltd., China). The needle was inserted into the cyst cavity, producing a sense of release as the gas escaped. Cystic gas and fluid were aspirated until the cyst wall visibly collapsed.

Observation group: After the above procedure, an additional intracapsular injection of anhydrous alcohol was performed without withdrawing the needle. The injection volume ranged from 0.2 to 0.5 mL, depending on the cyst size. For multiple lesions, aspiration and injection were repeated for each cyst sequentially or selectively for larger cysts. The mucosal surface was closely observed for color changes, bleeding, or perforation.

All procedures were performed by a single senior endoscopist with over 10 years of experience in therapeutic endoscopy to minimize operator bias.

Blinding of patients and operators was not feasible due to the visible procedural nature; this limitation is acknowledged.

#### Evaluation Criteria:

Patients were followed at 3, 6, and 12 months after treatment. If follow-up was incomplete, the most recent clinical assessment was used for analysis.

Therapeutic efficacy was categorized as follows:

Cured: Complete disappearance of cystic lesions on colonoscopy, resolution of abdominal symptoms, and negative stool occult blood.
Effective: Marked reduction in lesion size with partial symptom improvement.
Ineffective: No reduction in lesion size or improvement in symptoms.

Total effectiveness (%) = (Cured + Effective) / Total × 100

Patient satisfaction was assessed using a revised “Surgical Effectiveness Satisfaction Questionnaire,” adapted from validated patient-reported outcome measures used in gastrointestinal endoscopy. The tool consisted of 10 items scored from 0 to 100, with higher scores indicating greater satisfaction.

The internal consistency (Cronbach’s α = 0.92) and content validity index (0.85) confirmed acceptable psychometric reliability.

Satisfaction levels were categorized as:

- 90-100: Very satisfied
- 80-89: Satisfied
- <80: Dissatisfied

Total satisfaction rate (%) = (Very satisfied + Satisfied) / Total × 100.

### Statistical Analysis

Sample size was estimated from a pilot study assuming a 20% improvement in total effectiveness (α = 0.05, power = 0.8). This calculation yielded a minimum of 38 patients per arm; to compensate for possible attrition, 43 participants were recruited for each group.

All data analyses were conducted using SPSS version 27.0 (IBM SPSS Corp.; Armonk, NY, USA). Continuous variables are expressed as mean ± SD and compared between groups using the *t*-test. Categorical variables are expressed as counts and percentages, and compared using the *χ*^2^ test or Fisher’s exact test when appropriate. Effect sizes are reported as mean differences for continuous outcomes and absolute risk differences (ARDs) for categorical outcomes, each with 95% CIs. Analyses were performed on an intention-to-treat basis including all allocated participants; because there were no losses to follow-up, the intention-to-treat and per-protocol populations were identical. Any secondary endoscopic alcohol injection administered to control-group patients after the prespecified 3-month outcome assessment was considered rescue treatment and is reported descriptively; between-group comparisons were based on the initially allocated treatment (intention-to-treat). A two-tailed *P*-value <.05 was considered statistically significant.

## Results

### Baseline Information

A total of 86 patients (43 per group) were included in the intention-to-treat analysis. There were no losses to follow-up; therefore, the intention-to-treat and per-protocol populations were identical.

Baseline characteristics, including age, sex, body mass index (BMI), duration of illness, liver function tests aspartate aminotransferase [AST], alanine aminotransferase [ALT], ESR, CRP, carbohydrate antigen 19-9 [CA19-9], CEA, and AFP, demonstrated no significant differences between the groups (*P* > .05) (Table 1).

All patients were followed for at least 3 months. Those with longer follow-up had clinical and colonoscopic data, information, usually at 3 or 6 months, recorded for analysis.

### Treatment Effectiveness

In the observation group, 36 patients were cured and 7 showed improvement, yielding a total effectiveness rate of 100.00%.

In the control group, 30 patients were cured, 9 showed improvement, and 4 were ineffective, yielding a total effectiveness rate of 88.37%.

The total effectiveness rate differed significantly (*χ*^2^ = 5.31, *P* = .02) between the groups (Table 2). The ARD in total effectiveness was 11.63 percentage points (95% CI: 2.05-21.21).

Five control-group patients were classified as ineffective at the prespecified 3-month assessment after aspiration alone. After this assessment, they underwent a second endoscopic session with aspiration plus anhydrous alcohol injection as rescue therapy, with subsequent improvement and resolution the cystic lesions. For statistical analyses, these patients remained in the control group (intention-to-treat) and were counted as non-responders for the primary effectiveness outcome.

Sub-group analysis was performed by location (ascending colon, transverse colon, descending colon, sigmoid colon) and size of lesion; and the treatment response was similar by location (*P* > .05).

### Recurrence

During follow-up, the observation group experienced no recurrences; whereas 5 patients (11.63%) in the control group experienced recurrent cystic lesions, as shown by colonoscopy. The recurrence rate was significantly different (*χ*^2^ = 5.31, *P* = .02) between the groups (Table 3). The ARD in recurrence was −11.63 percentage points (95% CI: −21.21 to −2.05).

All recurrent cystic lesions and associated symptoms in the control group were successfully treated again with endoscopic aspiration and anhydrous alcohol injection after recurrence was documented. These re-treatments did not alter the recurrence analysis, which was based on recurrence status prior to retreatment.

### Patient Satisfaction

Overall satisfaction was significantly higher in the observation group (95.35%) than in the control group (79.07%), corresponding to an ARD of 16.28 percentage points (95% CI: 2.59-29.97).

The difference in satisfaction rate was significant (*χ*^2^ = 5.11, *P* = .02) between groups (Table 4).

The patients in the observation group reported better improvement of signs of abdominal discomfort, quicker relief of symptoms, and perceived a greater effectiveness of treatment.

### Postoperative Complications

All 86 colonoscopic procedures were accomplished as planned, and no immediate or short-term complications were observed.

No immediate perforation, bleeding, obstruction, or infection was observed in either group, supporting the short-term procedural safety of both endoscopic approaches. Longer follow-up is needed to assess the late adverse events.

## Discussion

Pneumatosis cystoides intestinalis is a rare gastrointestinal disorder that occurs with an incidence rate of roughly 0.03%-0.2% in the general population.^20^^-^^23^ The elevated cases of PCI at high-altitude locations such as Xinjiang, Qinghai, and Gansu imply that environmental or dietary factors may play a role in the regional incidence.^24^ This geographical pattern may help explain the relatively high number of patients enrolled in this study despite the rarity of the disease overall.

The present study demonstrated that endoscopic mucosal needle aspiration combined with intracapsular injection of anhydrous alcohol had a higher total effectiveness rate, a lower recurrence rate, and greater postoperative satisfaction compared to aspiration alone. These findings correspond with previous studies supporting endoscopic treatment as a safe and effective management of PCI. Le et al^25^ reported a single case successfully treated with aspiration. Wang et al^26^ reported successful symptom resolution in 6 patients after endoscopic treatment, although some recurred. Tran et al^27^ and Molina et al^28^ confirmed the feasibility, safety, and positive results of endoscopic treatment as a minimally invasive alternative to surgical intervention. This study adds to the literature by confirming that intracapsular alcohol injections promote cyst wall sclerosis, thereby decreasing recurrence.

Mechanistically, anhydrous alcohol affects chemical sclerosis of the cyst wall through protein denaturation, vascular constriction, and aseptic fibrosis.^29^ These processes cause degeneration and adhesion of the cystic lining, leading to persistent cavity closure and eventual gas resorption. This same mechanism accounts for the long-term benefits observed in the alcohol-treated group. Additionally, endoscopic therapy enables direct mechanical and chemical decompression of cysts with rapid onset and is associated with a lower recurrence rate than conservative measures such as hyperbaric oxygen, antibiotics, or bowel rest.^30^^-^^33^

Of note, this therapy is clinically straightforward and minimally invasive, making it a viable option for a subset of patients who would otherwise be surgical candidates but are limited by age, comorbidities, or poor general health. The procedure allows direct visualization of the lesion, followed by aspiration under direct vision and controlled injections into specific localized target areas which were all performed with minimal tissue trauma and a low risk of complications. In all reported cases, there were no instances of perforation, hemorrhage, obstruction, or infection, further supporting the high procedural safety profile previously established.^19^^,^^20^^,^^34^^-^^36^

The study had several limitations. First, this was a single-center study, which limits generalizability. Second, due to the visible nature of the procedures, blinding was not performed. Third, the study was not prospectively registered and allocation was quasi-random, which may introduce selection and performance bias. Fourth, the total sample size and the fact that all procedures were performed by a single operator may limit reproducibility. Finally, the ≤12-month follow-up may not fully capture late adverse events or delayed complications (including late recurrence), therefore longer follow-up is needed.

Future studies on this topic should consider a multicenter prospective trial design with a larger sample size to further validate these results and develop evidence for optimal procedural-specific methods. Future studies should also extend the follow-up period to assess late recurrences and long-term outcomes. Next, some form of cost-effectiveness analysis and/or standardized selection criteria for eligible patient selection would also be critically important for an optimal procedural framework to define the role of alcohol-assisted endoscopic therapy for colonic PCI.

In conclusion, endoscopic mucosal needle aspiration combined with intracapsular anhydrous alcohol injection appeared feasible and was not associated with immediate or short-term complications in this single-center cohort. Although short-term outcomes favored the combined approach, the quasi-randomized design and limited follow-up preclude definitive conclusions regarding clinical superiority. Multicenter studies with longer follow-up are warranted.

## Figures and Tables

**Table 1.** Baseline Characteristics of the Participants

**Parameters**	**Observation group (n = 43)**	**Control group (n = 43)**	***t*/*χ*** **^2^**	***P***
Age (years)	44.77 ± 5.22	44.28 ± 3.72	0.500	.619
Gender (Female/Male)	12/31	10/33	0.244	.621
BMI (kg/m^2^)	23.56 ± 2.14	23.38 ± 2.05	0.405	.687
Duration of disease (years)	1.62 ± 0.31	1.59 ± 0.28	0.499	.619
AST (U/L)	18.29 ± 2.96	17.64 ± 3.36	0.946	.347
ALT (U/L)	22.35 ± 3.64	23.18 ± 4.26	0.967	.336
Erythrocyte sedimentation rate (mm/h)	8.92 ± 2.06	9.04 ± 2.31	0.247	.805
C-reactive protein (mg/L)	4.91 ± 1.37	4.78 ± 1.53	0.412	.681
CA19-9 (IU/L)	24.08 ± 7.16	23.59 ± 7.33	0.310	.757
Carcinoembryonic antigen (μg/L)	1.64 ± 0.74	1.57 ± 0.81	0.447	.656
Alpha-fetoprotein (ug/L)	10.86 ± 2.62	11.24 ± 2.14	0.737	.463

Data are mean ± SD or n (%). Continuous variables were compared using the *t*-test, and categorical variables using the *χ*^2^ test.

BMI, body mass index; AST, aspartate aminotransferase; ALT, alanine aminotransferase; CA19-9, carbohydrate antigen 19-9.

Data are mean ± SD or n (%).

**Table 2.** Therapeutic Effectiveness at 3 Months

**Treatment Response**	**Observation Group (n = 43), n (%)**	**Control Group (n = 43), n (%)**	***χ*** **^2^**	***P***	**Absolute Risk Difference (95% CI)**
Cured	36 (83.72)	30 (69.77)			
Effective	7 (16.28)	8 (18.60)			
Ineffective	0 (0)	5 (11.63)			
Total effectiveness rate	43 (100)	38 (88.37)	5.309	.021	+11.63% (95% CI: 2.05%-21.21%)

*χ*^2^ and *P*-values refer to the between-group comparison of total effectiveness. Effect size is reported as absolute risk difference (ARD) with 95% CI.

**Table 3.** Postoperative Recurrence Within 12 Months of Follow-Up

**Groups**	**Postoperative Recurrence**	**Absolute Risk Difference (95% CI)**
Observation group (n = 43)	0 (0%)	
Control group (n = 43)	5 (11.63%)	
*χ* ^2^	5.309	−11.63% (95% CI: −21.21% to −2.05%)
*P*	.021	

*χ*^2^ test was used for between-group comparison. Effect size is reported as absolute risk difference with 95% CI.

**Table 4.** Patient Satisfaction After Treatment

**Clinical Response**	**Observation Group (n = 43), n (%)**	**Control Group (n = 43), n (%)**	***χ*** **^2^**	***P***	**Absolute Risk Difference (95% CI)**
Satisfied	33 (76.74)	21 (48.84)			
Partially satisfied	8 (18.60)	13 (30.23)			
Dissatisfied	2 (4.65)	9 (20.93)			
Total satisfaction	41 (95.35)	34 (79.07)	5.108	.024	+16.28% (95% CI: 2.59%-29.97%)

*χ*^2^ and *P*-values refer to the between-group comparison of total satisfaction. Effect size is reported as absolute risk difference (ARD) with 95% CI.

## Data Availability

The data that support the findings of this study are available on request from the corresponding author.
